# A PI3-Kinase–Mediated Negative Feedback Regulates Neuronal Excitability

**DOI:** 10.1371/journal.pgen.1000277

**Published:** 2008-11-28

**Authors:** Eric Howlett, Curtis Chun-Jen Lin, William Lavery, Michael Stern

**Affiliations:** Department of Biochemistry and Cell Biology, Rice University, Houston, Texas, United States of America; The Jackson Laboratory, United States of America

## Abstract

Use-dependent downregulation of neuronal activity (negative feedback) can act as a homeostatic mechanism to maintain neuronal activity at a particular specified value. Disruption of this negative feedback might lead to neurological pathologies, such as epilepsy, but the precise mechanisms by which this feedback can occur remain incompletely understood. At one glutamatergic synapse, the Drosophila neuromuscular junction, a mutation in the group II metabotropic glutamate receptor gene (*DmGluRA*) increased motor neuron excitability by disrupting an autocrine, glutamate-mediated negative feedback. We show that *DmGluRA* mutations increase neuronal excitability by preventing PI3 kinase (PI3K) activation and consequently hyperactivating the transcription factor Foxo. Furthermore, glutamate application increases levels of phospho-Akt, a product of PI3K signaling, within motor nerve terminals in a *DmGluRA*-dependent manner. Finally, we show that PI3K increases both axon diameter and synapse number via the Tor/S6 kinase pathway, but not Foxo. In humans, PI3K and group II mGluRs are implicated in epilepsy, neurofibromatosis, autism, schizophrenia, and other neurological disorders; however, neither the link between group II mGluRs and PI3K, nor the role of PI3K-dependent regulation of Foxo in the control of neuronal excitability, had been previously reported. Our work suggests that some of the deficits in these neurological disorders might result from disruption of glutamate-mediated homeostasis of neuronal excitability.

## Author Summary

Use-dependent downregulation of neuronal excitability (negative feedback) can act to maintain neuronal activity within specified levels. Disruption of this homeostasis can lead to neurological disorders, such as epilepsy. Here, we report a novel mechanism for negative feedback control of excitability in the Drosophila larval motor neuron. In this mechanism, activation by the excitatory neurotransmitter glutamate of metabotropic glutamate receptors (mGluRs) located at motor nerve terminals decreases excitability by activating PI3 kinase (PI3K), consequently causing the phosphorylation and inhibition of the transcription factor Foxo. Foxo inhibition, in turn, decreases neuronal excitability. These observations are of interest for two reasons. First, our observation that PI3K activity regulates neuronal excitability is of interest because altered PI3K activity is implicated in a number of neurological disorders, such as autism and neurofibromatosis. Our results raise the possibility that altered excitability might contribute to the deficits in these disorders. Second, our observation that group II metabotropic glutamate receptors (mGluRs) activate PI3K is of interest because group II mGluRs are implicated in epilepsy, anxiety disorders, and schizophrenia. Yet the downstream signaling pathways affected by these treatments are incompletely understood. Our results raise the possibility that the PI3K pathway might be an essential mediator of signalling by these mGluRs.

## Introduction

Negative feedback processes, which can enable maintenance of neuronal homeostasis, are widely observed in neuronal systems [1]–[3]. For example, neuronal silencing via tetrodotoxin application both *in vivo* and *in vitro* increases excitability [4]–[6]. This effect occurs *in vitro* via both increased sodium currents and decreased potassium currents. However, the signaling pathways responsible for these excitability changes remain unclear.

The mammalian group II metabotropic glutamate receptors, which are G-protein coupled receptors activated by glutamate, are well positioned to mediate negative feedback. When localized presynaptically, these receptors can act as autoinhibitors of glutamate release [7]–[10]. Because these receptors are located outside of the active zone [11], activation is thought to occur only during conditions of elevated glutamate release and might serve to prevent glutamate-mediated neurotoxicity. Agonists for these receptors are proposed for treatment of schizophrenia, anxiety and epilepsy, among others [12],[13], but the mGluR-dependent signaling pathways that underlie these disorders remain unidentified. Furthermore, although many of the acute effects of group II mGluR activation on neuronal physiology have been elucidated [14],[15], possible long term effects on neuronal function, such as through changes in ion channel gene expression, remain essentially unexplored.

In Drosophila, the single *DmGluRA* gene encodes a protein most similar to the mammalian group II mGluR [16]. DmGluRA is located presynaptically at the neuromuscular junction (nmj), which suggests that DmGluRA might regulate transmitter release from motor neurons. Elimination of *DmGluRA* by the null mutation *DmGluRA^112b^*, or by RNAi-mediated *DmGluRA* knockdown specifically in motor neurons, increases neuronal excitability [16]. Given that glutamate is the excitatory neurotransmitter from Drosophila motor neurons, the increased excitability of *DmGluRA* mutants raised the possibility that DmGluRA decreases motor neuron excitability upon activation by glutamate released from motor nerve terminals. In this view, DmGluRA would mediate an activity-dependent negative feedback on excitability. However, the mechanism by which this negative feedback is accomplished was not elucidated.

Here we show that glutamate-mediated activation of DmGluRA decreases neuronal excitability by activating the lipid kinase PI3 kinase (PI3K), which promotes growth and inhibits apoptosis in various cell types. In particular, we report that transgene-induced inhibition of PI3K in motor neurons confers neuronal excitability phenotypes similar to *DmGluRA^112b^*, whereas transgene-induced activation of PI3K confers the opposite excitability phenotypes. We also show that PI3K activation in motor neurons suppresses the increased excitability of *DmGluRA^112b^*, and glutamate application to motor nerve terminals activates PI3K in a DmGluRA-dependent manner. Finally, we show that altered PI3K activity regulates both axon diameter and synapse number, and that these effects on neuronal growth are mediated by the Tor/S6 kinase pathway, whereas the effects of PI3K on neuronal excitability are mediated by the transcription factor Foxo. We conclude that negative feedback of Drosophila motor neuron excitability occurs via the glutamate-induced activation of DmGluRA autoreceptors, causing the PI3K-dependent inhibition of Foxo and a consequent decrease in neuronal excitability. A similar negative feedback operating in the mammalian CNS might underlie neuronal disorders involving the group II mGluRs or PI3K.

## Results

### Drosophila mGluRA (DmGluRA) Affects the Rate of Onset of Long-Term Facilitation (LTF), a Reporter for Motor Neuron Excitability

**Figure 1:**
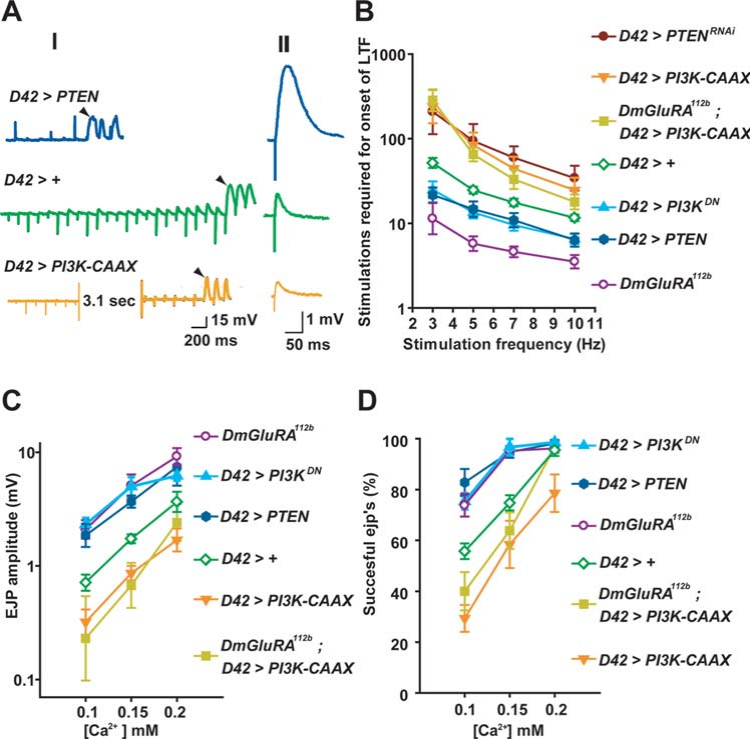
DmGluRA activity inhibits neuronal excitability via activation of the PI3K pathway. The motor neuron GAL4 driver *D42* was used to drive expression of all transgenes. For all LTF experiments, the bath solution contained 0.15 mM [Ca^2+^] and 100 µM quinidine, which is a K^+^ channel blocker that sensitizes the motor neuron and enables LTF to occur and measured even in hypoexcitable neurons. A) Representative traces showing the decreased rate of onset of long-term facilitation (LTF) (I) and decreased excitatory junction potential (ejp) amplitude (II) in larvae overexpressing *PI3K-CAAX* in motor neurons compared to wildtype at the indicated [Ca^2+^], and the increased rate of onset of LTF and ejp amplitude in larvae overexpressing *PTEN*. Arrowheads indicate the increased and asynchronous ejps, indicative of onset of LTF. In (II), ejps are averages of 180 responses for each genotype. B) Number of stimulations required to induce LTF (Y axis) at the indicated stimulus frequencies (X axis) in the indicated genotypes. Geometric means+/−SEMs are shown. From top to bottom, *n* = 6, 12, 7, 18, 12, 21, and 6 respectively, for each genotype. One-way ANOVA and Fisher's LSD gave the following differences, at 10 Hz, 7 Hz, 5 Hz and 3 Hz, respectively: For *D42>+*: vs. *D42>PI3K-CAAX*, p = 0.013, 0.0021, 0.0002, <0.0001; vs. *D42*>PTEN, p = 0.011, 0.056, 0.079, 0.0054; vs. *D42>PTEN^RNAi^*, p = 0.0018, 0.0004, 0.0006, 0.0014; vs. *D42>PI3K^DN^*, p = 0.035, 0.036, 0.05, 0.038; vs. *mGluR^112b^*, p = 0.0012, 0.0005, 0.0004, 0.0009. For *mGluR^112b^*, *D42>PI3K-CAAX* vs: *mGluR^112b^*, p = 0.0003, <0.0001, <0.0001, <0.0001; vs. *D42>PI3K-CAAX*, p = 0.33, 0.34. 0.46, 0.62. C) Mean ejp amplitudes (Y axis) at the indicated [Ca^2+^] (X axis), from the indicated genotypes. Larval nerves were stimulated at a frequency of 1 Hz, and 10 responses were measured from each of nine larvae (for *D42>PI3K-CAAX* and *D42>PI3K^DN^*) and for six larvae from other genotypes. Means+/−SEMs are shown. One-way ANOVA and Fisher's LSD gave the following differences, at 0.1 mM, 0.15 mM and 0.2 mM [Ca^2+^], respectively: For *D42>+*: vs. *D42>PI3K-CAAX*, p = 0.028, 0.05, 0.04; vs. *D42*>PTEN, p = 0.017, 0.03, 0.06; vs. *D42>PI3K^DN^*, p = 0.0018, 0.0033, 0.14; vs. *mGluR^112b^*, p = 0.0077, 0.0029, 0.01. For *mGluR^112b^*, *D42>PI3K-CAAX* vs: *mGluR^112b^*, p<0.0001, <0.0001, 0.0001; vs. *D42>PI3K-CAAX*, p = 0.70, 0.47, 0.26. D) Effects of altered PI3K pathway activity on failures of transmitter release. Mean transmitter release success rate +/−SEMs (Y axis) at the indicated Ca^2+^ concentration (X axis) for the indicated genotypes. Larval nerves were stimulated at 1 Hz. 10 responses were collected per nerve from each of 6 larvae for the given genotype and at the given Ca^2+^ concentration. One-way ANOVA and Fisher's LSD gave the following differences, at 0.1 mM, 0.15 mM and 0.2 mM [Ca^2+^], respectively: For *D42>+*: vs. *D42>PI3K-CAAX*, p = 0.0023, 0.023, 0.001; vs. *D42>*PTEN, p = 0.0014, 0.0068, 0.69; vs. *D42>PI3K^DN^*, p = 0.011, 0.003, 0.63; vs. *mGluR^112b^*, p = 0.027, 0.0053, 0.99. For *mGluR^112b^*, *D42>PI3K-CAAX* vs: *mGluR^112b^*, p = 0.0001, <0.0001, 0.94; vs. *D42>PI3K-CAAX*, p = 0.21, 0.45, 0.0008.

The increase in neuronal excitability conferred by the *DmGluRA^112b^* null mutation is manifested by an increased rate of onset of a form of synaptic plasticity termed long-term facilitation (LTF) [16],[17], which is induced when a motor neuron is subjected to repetitive nerve stimulation at low bath [Ca^2+^]. At a certain point in the stimulus train, an abrupt increase in transmitter release and hence muscle depolarization (termed excitatory junctional potential, or ejp) is observed (Figure 1A). LTF not only increases ejp amplitude, but also ejp duration, indicative of prolonged and asynchronous transmitter release (Figure 1A). This abrupt increase in the amount and duration of transmitter release is caused by an abrupt increase in the duration of nerve terminal depolarization and hence Ca^2+^ influx, and reflects a progressive increase in motor neuron excitability induced by the repetitive nerve stimulation: when an excitability threshold is reached, LTF occurs [17]–[19].

In Drosophila, many genotypes that increase motor neuron excitability by decreasing K^+^ currents or increasing Na^+^ currents increase the rate of onset of LTF. For example, altered activities of *frequenin* and *Hyperkinetic*, which act via K^+^ channels, or *paralytic* and *pumilio*, which act via Na^+^ channels, each increase the rate of onset of LTF [18]–[24]. By increasing motor neuron excitability, the genotypes described above apparently bring excitability closer to the threshold required to evoke LTF and consequently decrease the number of prior nerve stimulations required to reach this threshold. In these genotypes, the prolonged nerve terminal depolarizations that triggered LTF were revealed by recording ejps and simultaneously recording extracellularly electrical activity within the peripheral nerves during LTF onset. It was found that LTF onset was accompanied by the appearance within peripheral nerves of supernumerary action potentials occurring at about 10 msec intervals following the initial induced action potential [18]–[25]. Several lines of evidence suggested that these supernumerary action potentials arose in motor axons and were responsible for the increased transmitter release underlying LTF. First, the number of these supernumerary action potentials correlated with ejp duration, and second, these supernumerary action potentials often preceded depolarizing steps in the asynchronous, multi-step ejps that occurred after LTF onset. Similar supernumerary action potentials were observed following nerve stimulation in the *eag Sh* double mutant, in which two distinct K channel α subunits are simultaneously eliminated, and which consequently exhibits extreme neuronal hyperexcitability. In the *eag Sh* double mutant, these supernumerary action potentials arise in the motor nerve terminals and exhibit retrograde propagation [25]. It was suggested that the supernumerary action potentials were caused by, and also prolonged, motor nerve terminal depolarization, and thus participated in a positive feedback loop prolonging depolarization [25]. This positive feedback loop presumably underlies the abrupt, threshold-like onset of LTF.

The observation that *mGluR^112b^* increases the rate of onset of LTF suggested that *DmGluRA^112b^* increases motor neuron excitability as well [16]. To confirm this suggestion, we simultaneously recorded peripheral nerve electrical activity and ejps during LTF induced by 10 Hz stimulus trains. As previously observed in the hyperexcitable genotypes described above [18]–[25], we found that the abrupt onset of LTF in *mGluRA^112b^* was accompanied in the nerve by the appearance of supernumerary action potentials (Figure S1). This observation confirmed that LTF onset in *mGluRA^112b^* was caused by prolonged motor nerve terminal depolarization, and hence that *mGluRA^112b^* increases neuronal excitability. Thus, as suggested previously [16], it appears that DmGluRA mediates an activity-dependent inhibition of neuronal excitability. In this view, glutamate release from motor nerve terminals downregulates subsequent neuronal activity by activating presynaptic DmGluRA autoreceptors, which then decrease excitability. Elimination of DmGluRA disrupts this negative feedback and prevents the decrease in excitability from occurring.

### The *DmGluRA^112b^*-Null Mutation Increases Neuronal Excitability by Preventing PI3K Activation

In addition to increasing neuronal excitability, *DmGluRA^112b^* also decreases arborization and synapse number at the larval neuromuscular junction [16]. This phenotype is also observed in larval motor neurons with decreased activity of PI3K [26]. This observation raised the possibility that DmGluRA might exert its effects on neuronal excitability as well as synapse formation via PI3K activity. To test the possibility that PI3K mediates the effects of DmGluRA on neuronal excitability, we used the *D42 Gal4* driver [27],[28] to overexpress transgenes expected to alter activity of the motor neuron PI3K pathway. We found that inhibiting the PI3K pathway by motor neuron-specific overexpression of either the phosphatase *PTEN*, which opposes the effect of PI3K, or the dominant-negative *PI3K^DN^* [29], each significantly increased the rate of onset of LTF, similarly to that of *DmGluRA^112b^* (Figure 1A and 1B). In contrast, we found that activating the PI3K pathway by expression of the constitutively active *PI3K-CAAX* [29], or via RNAi-mediated inhibition of *PTEN*, decreased rate of onset of LTF (Figure 1A and 1B). As was described above for *mGluRA^112b^*, LTF onset was accompanied by the appearance of supernumerary action potentials in the nerve (Figure S1) demonstrating that altered excitability is responsible for the altered rate of onset of LTF in these genotypes.

The rate of LTF onset described above was measured in the presence of the potassium channel blocking drug quinidine, which moderately increases neuronal excitability and hence rate of onset of LTF in the larval motor neuron. Quinidine application sensitizes the motor neuron to the effects of the nerve stimulation and enables LTF to occur reliably in genotypes with low excitability, even at lower stimulus frequencies. To demonstrate that altered PI3K activity does not alter rate of onset of LTF by altering sensitivity to quinidine, we compared the timing of LTF onset in the absence of quinidine in wildtype larvae and in larvae with inhibited PI3K. We found that inhibiting PI3K activity in motor neurons significantly accelerated LTF onset even in the absence of quinidine (Figure S2) demonstrating that altered sensitivity of motor neurons to quinidine does not underlie the altered onset rate of LTF that we observe.

In addition to effects on LTF, mutations that alter motor neuron excitability can alter basal transmitter release and hence ejp amplitude at low bath Ca^2+^ concentrations, at which Ca^2+^ influx would be limiting for vesicle fusion to occur. For example, mutations in *ether-a go-go (eag)*, which encodes a potassium channel α subunit, increase transmitter release about two-fold [25], whereas a mutation in the sodium channel gene *paralytic* decreases transmitter release by increasing the frequency at which nerve stimulation failed to evoke any vesicle fusion, termed “failure” of vesicle release [30]. Presumably altered excitability affects the amplitude or duration of the action potential and consequently the amount of Ca^2+^ influx through voltage-gated channels. We found that *DmGluRA^112b^* also increased ejp amplitude and hence basal transmitter release at three low bath Ca^2+^ concentrations tested (Figure 1C), which is consistent with increased motor neuron excitability in this genotype. We found that decreasing PI3K pathway activity via motor neuron overexpression of *PI3K^DN^* or *PTEN* also increased transmitter release to levels similar to *DmGluRA^112b^*, whereas increasing PI3K pathway activity via overexpression of *PI3K-CAAX* decreased basal transmitter release (Figure 1C).

The *DmGluRA^112b^* mutation also decreased the frequency at which failures of vesicle release occur, particularly at the lower Ca^2+^ concentrations tested (Figure 1D). This observation confirms that the effect of *DmGluRA^112b^* on ejp amplitude is presynaptic. We also observed a decreased frequency of failures when the PI3K pathway was inhibited by motor neuron expression of *PI3K^DN^* or *PTEN* (Figure 1D). In contrast, motor neuron overexpression of *PI3K-CAAX* increased the frequency of failures (Figure 1D). Therefore, with three electrophysiological readouts, the *DmGluRA^112b^* mutant phenotype was mimicked by decreased activity of the PI3K pathway, whereas increasing PI3K pathway activity conferred opposite effects.

These observations support the notion that loss of DmGluRA increases motor neuron excitability by preventing the activation of PI3K. If so, then motor neuron expression of *PI3K-CAAX*, which will be active independently of DmGluRA, is predicted to suppress the *DmGluRA^112b^* hyperexcitability. To test this possibility, we drove motor-neuron expression of *PI3K-CAAX* in a *DmGluRA^112b^* background and found a rate of onset of LTF and ejp amplitude that was very similar to what was observed when *PI3K-CAAX* was expressed in a wildtype background, but significantly different from *DmGluRA^112b^* (Figure 1B, Figure 1C). In addition, motor neuron-specific expression of *PI3K-CAAX* increased failure frequency at the two lower [Ca^2+^] tested to the same level in *DmGluRA^112b^* larvae as in wildtype (Figure 1D). We conclude that hyperexcitability of the *DmGluRA^112b^* mutant results from inability to activate PI3K.

### Glutamate Application Increases Levels of Phosphorylated Akt in Motor Nerve Terminals in a DmGluRA-Dependent Fashion

**Figure 2:**
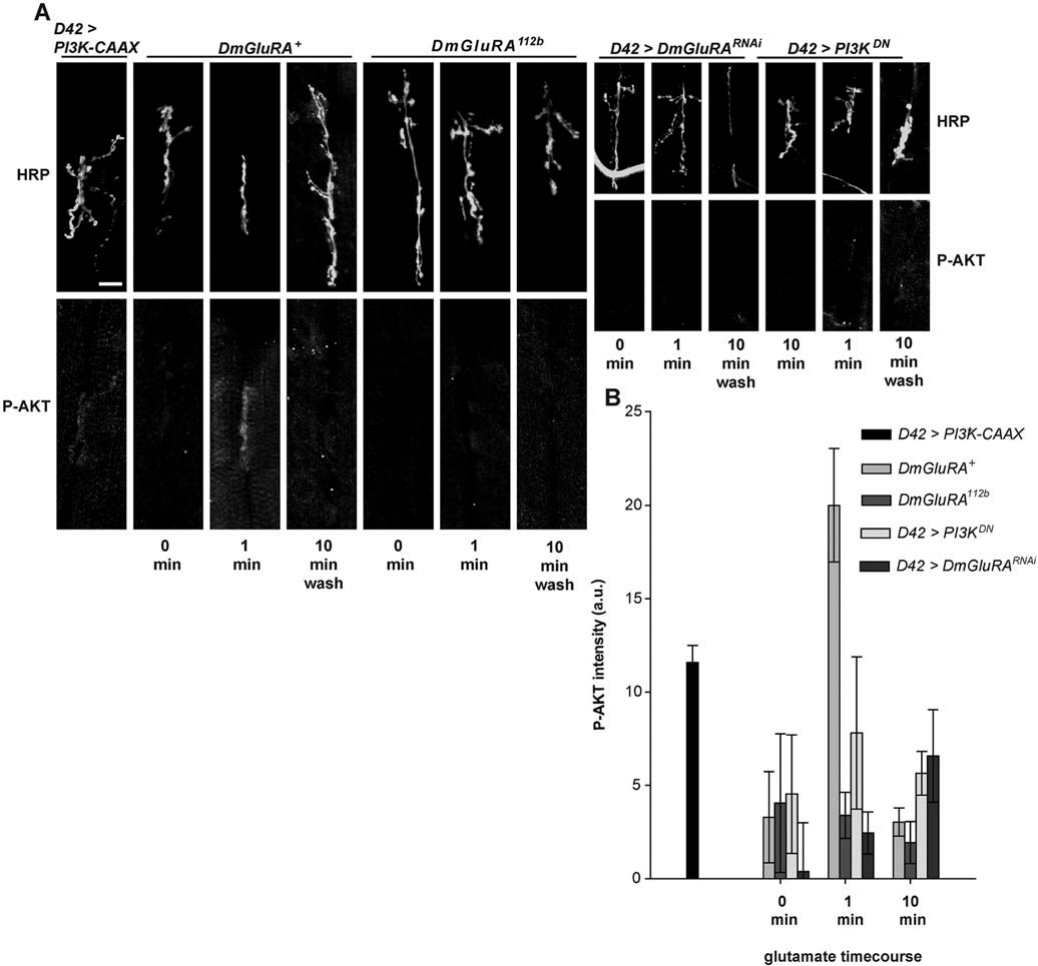
Glutamate application stimulates presynaptic Akt phosphorylation in *DmGluRA^+^* but not in *DmGluRA^112b^* mutant larvae. A) Representative confocal images of *DmGluRA^+^*, *DmGluRA^112b^*, *D42>DmGluRA^RNAi^* and *D42>PI3K^DN^* larvae stained with anti-HRP (upper) and anti-p-Akt (lower) in the indicated conditions. All images are from muscles 7 and 6 of abdominal segment A3 or A4. Scale bar = 20 µm. B) Quantification of phosphorylated Akt (p-Akt) levels in *DmGluRA^+^*, *DmGluRA^112b^*, *D42>DmGluRA^RNAi^* and *D42>PI3K^DN^* larvae immediately prior to glutamate application, after 1 min of 100 µM glutamate application (final bath concentration), and 10 min after a wash with glutamate free media. Nerve terminals were outlined with HRP fluorescence as reference. Pixel intensities were quantified using ImageJ software and background subtraction was performed as described in detail in Methods section. Bars represent mean synaptic p-Akt levels +/−SEMs. *D42>PI3K-CAAX* is included as a positive control. One-way ANOVA and Fisher's LSD gave the following significant differences for p-Akt levels one minute after glutamate application. For *DmGluRA^+^* vs. *DmGluRA^112b^*, p = 0.0072; vs. *D42>PI3K^DN^*, p = 0.0097; vs. *D42>DmGluRA^RNAi^*, p<0.0001.

The results described above suggest that glutamate release from motor nerve terminals as a consequence of motor neuron activity activates PI3K within motor nerve terminals via DmGluRA autoreceptors. To test this possibility directly, we measured the ability of glutamate applied to the neuromuscular junction to activate PI3K within motor nerve terminals. To assay for PI3K activity we applied an antibody specific for the phosphorylated form of the kinase Akt (p-Akt), which is increased by elevated PI3K pathway activity. The usefulness of this antibody for specific detection of Drosophila p-Akt has been previously demonstrated [31]–[33]. The ability to detect p-Akt in larval motor nerve terminals overexpressing *PI3K-CAAX*, but not in wildtype (Figure 2), further validates this antibody as a PI3K reporter.

We compared p-Akt levels in wildtype versus *DmGluRA^112b^* motor nerve terminals immediately prior to or following a 1 minute application of 100 µM glutamate. We found that glutamate application strongly increased p-Akt levels in wildtype larvae, but not in the *DmGluRA^112b^* larvae (Figure 2), demonstrating that glutamate application increases nerve terminal p-Akt levels, and that DmGluRA activity is required for this increase.

We found that DmGluRA activity was required presynaptically for this p-Akt increase: motor neuron-specific expression of a *DmGluRA* RNAi construct [16], blocked the ability of glutamate to increase p-Akt levels (Figure 2). In [16] it was reported that expression of *DmGluRA* RNAi decreased, but did not eliminate, mGluRA immunoreactivity, suggesting that this transgene decreases, but does not eliminate, glutamate-mediated signalling via mGluRA. The ability of this transgene to block glutamate-mediated induction of p-Akt suggests that activation of PI3K by glutamate is sensitive to mGluRA levels and requires a minimum level of mGluRA expression. In contrast, expression of the *DmGluRA* RNAi construct in the muscle, with use of the *24B Gal4* driver, did not inhibit p-Akt levels: p-Akt intensity following 1 minute of glutamate application was not significantly different from wildtype (17.6+/−2.9, p = 0.59).

To determine if PI3K activity was required presynaptically for this glutamate-induced p-Akt increase, we inhibited PI3K activity by motor neuron-expression of *PI3K^DN^*, and found that this transgene significantly inhibited the ability of glutamate to activate p-Akt (Figure 2). Thus, presynaptic DmGluRA and PI3K activity are both necessary for glutamate to increase p-Akt.

### The Effects of PI3K on Neuronal Excitability Are Mediated by Foxo, not Tor/S6 Kinase

Many effects of the PI3K pathway are mediated by the downstream kinase Akt. Activated Akt phosphorylates targets such as Tsc1/Tsc2, which regulates cell growth via the Tor/S6 Kinase (S6K) pathway [34], Foxo, which regulates apoptosis [35], and GSK3 [36], which mediates at least in part the effects of altered PI3K pathway activity on arborization and synapse number [26]. All of these Akt-mediated phosphorylation events inhibit activity of the target protein.

**Figure 3:**
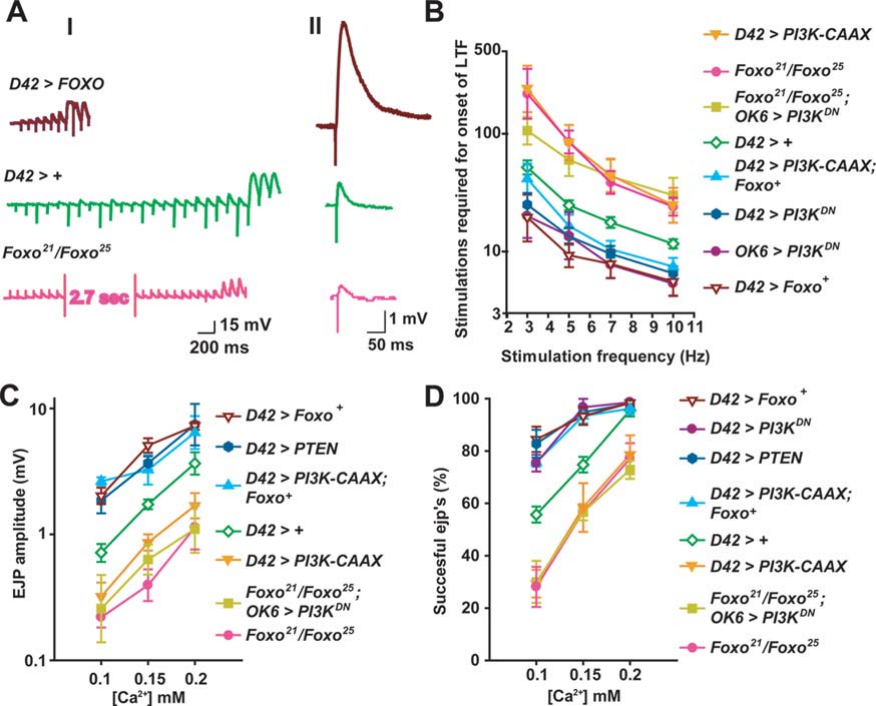
*Foxo* mediates the effects of *PI3K* on motor neuron excitability. The *Gal4* driver *D42* was used to drive expression of transgenes in all genotypes except for *Foxo^21^/Foxo^25^*; *OK6>PI3K^DN^*, in which the motor neuron driver *OK6* was used and which behaves similarly to *D42* in this assay. For all LTF experiments, the bath solution contained 0.15 mM [Ca^2+^] and 100 µM quinidine. A) Representative traces showing the decreased rate of onset of LTF (I) and decreased ejp amplitude (II) in *Foxo^21^/Foxo^25^* larvae compared to wildtype at the indicated [Ca^2+^], and the increased rate of onset of LTF and ejp amplitude in larvae overexpressing *Foxo*. Arrowheads indicate the increased and asynchronous ejps, indicative of onset of LTF. In (II), representative traces are averages of multiple ejps. From *top* to *bottom*, *n* = 23, 180, and 34 respectively. B) Number of stimulations required to induce LTF (Y axis) at the indicated stimulus frequencies (X axis). Geometric means+/−SEMs are shown. From *top* to *bottom*, *n* = 12, 6, 7, 18, 10, 21, 5, and 9 respectively, for each genotype. One-way ANOVA and Fisher's LSD gave the following differences, at 10 Hz, 7 Hz, 5 Hz and 3 Hz, respectively: For *D42>+* vs. *Foxo^21^/Foxo^25^*, p = 0.0096, 0.0069, <0.0001, 0.0007; vs. *D42>Foxo^+^*, p = 0.0026, 0.0012, <0.0001, 0.0065. For *D42>PI3K-CAAX*, *Foxo* vs. *D42>PI3K-CAAX*, p = 0.0041, 0.0005, 0.0002, 0.0006; vs. *D42>Foxo*, p = 0.50, 0.43, 0.16, 0.14. For *Foxo^21^Foxo^25^*; *OK6>PI3K^DN^* vs. *OK6>PI3K^DN^*, p = ; 0.0003, 0.0004, 0.0014, 0.001. vs. *Foxo^21^Foxo^25^*, p = 0.63, 0.74, 0.43, 0.20. C) Mean ejp amplitude +/−SEMs (Y axis) for each genotype at the indicated [Ca^2+^]. Nerves from six larvae were stimulated at a frequency of 1 Hz, and 10 responses were measured per larva. One-way ANOVA and Fisher's LSD gave the following differences, at 0.1 mM, 0.15 mM and 0.2 mM [Ca^2+^], respectively: For *D42>+* vs. *Foxo^21^/Foxo^25^*, p = 0.0079, <0.0001, 0.012; vs. *D42>Foxo*, p = 0.017, 0.0005, 0.10. For *Foxo^21^/Foxo^25^*; *OK6>PI3K^DN^*: vs. *Foxo^21^/Foxo^25^*, p = 0.74, 0.12, 0.93; vs. *D42>PI3K^DN^*, p<0.0001, <0.0001, 0.0001; vs. *D42>PTEN*, p<0.0001, <0.0001, <0.0001. For *D42>PI3K-CAAX*, *Foxo* vs. *D42>PI3K-CAAX*, p<0.0001, <0.0001,  = 0.0024; vs. *D42>Foxo*, p = 0.52, 0.13, 0.77. D) Mean transmitter release success rate +/−SEMs (Y axis) at the indicated Ca^2+^ concentration (X axis) for the indicated genotypes. Larval nerves were stimulated at 1 Hz. 10 responses were collected per nerve from each of 6 larvae for the given genotype and at the given Ca^2+^ concentration. One-way ANOVA and Fisher's LSD gave the following differences, at 0.1 mM, 0.15 mM and 0.2 mM [Ca^2+^], respectively: For *D42>+* vs. *Foxo^21^/Foxo^25^*, p = 0.0008, 0.0039, 0.0009; vs. *D42>Foxo*, p = 0.0008, 0.004, 0.7. For *Foxo^21^/Foxo^25^*; *OK6>PI3K^DN^*: vs. *Foxo^21^/Foxo^25^*, p = 0.81, 0.99, 0.43. vs. *D42>PI3K^DN^*, p<0.0001, <0.0001, <0.0001. vs. *D42>PTEN*, p<0.0001, <0.0001. <0.0001. For *D42>PI3K-CAAX*, *Foxo* vs. *D42>PI3K-CAAX*, p<0.0001, <0.0001,  = 0.002; vs. *D42>Foxo*, p = 0.29, 0.98, 0.7.

If PI3K pathway activity decreases neuronal excitability by inhibiting Foxo, then Foxo overexpression is predicted to mimic the hyperexcitability observed when PI3K pathway activity is blocked in motor neurons, whereas loss of Foxo is predicted to mimic the hypoexcitability observed when *PI3K-CAAX* is expressed in motor neurons. To test these predictions, we measured neuronal excitability in larvae carrying the heteroallelic *Foxo^21^/Foxo^25^* null mutant combination [37] and in larvae overexpressing *Foxo^+^* [38] in motor neurons. We found that overexpression of *Foxo^+^* increased the rate of onset of LTF, basal transmitter release and frequency of successful ejps to a level very similar to that observed when PI3K pathway activity was decreased (Figure 3) whereas in *Foxo^21^/Foxo^25^* larvae, the rate of onset of LTF, basal transmitter release and frequency of successful ejps were decreased to levels very similar to those observed when *PI3K-CAAX* was expressed in motor neurons (Figure 3). These observations support the notion that PI3K activity decreases excitability by downregulating Foxo activity.

If the hyperexcitability conferred by motor neuron expression of *PI3K^DN^* results from Foxo hyperactivity, then the *Foxo^21^/Foxo^25^* null combination will suppress this hyperexcitability and confer motor neuron hypoexcitability similar to what is observed in *Foxo^21^/Foxo^25^* larvae in an otherwise wildtype background. We confirmed this prediction: larvae carrying the *Foxo^21^/Foxo^25^* null combination and expressing *PI3K^DN^* in motor neurons exhibited a rate of onset of LTF, basal transmitter release, and failure frequency very similar to what was observed in the *Foxo^21^/Foxo^25^* null mutant alone (Figure 3), or in larvae expressing *PI3K-CAAX* in motor neurons. We used the *OK6* motor neuron *Gal4* driver rather than *D42* for ease of stock construction in experiments involving *Foxo^21^/Foxo^25^*. *OK6* confers motor neuron phenotypes indistinguishable from *D42* in our assays (Figure 3B and not shown).

In addition, if the hypoexcitability conferred by motor neuron expression of *PI3K-CAAX* results from decreased Foxo activity, then co-overexpression of *Foxo^+^* will suppress this hypoexcitability and confer hyperexcitability similar to what is observed when *PI3K^DN^*, *PTEN* or *Foxo^+^* alone are expressed in motor neurons. We confirmed this prediction: larvae co-expressing *Foxo^+^* and *PI3K-CAAX* in motor neurons exhibited rate of onset of LTF, basal transmitter release and failure frequency very similar to what was observed when *PI3K^DN^*, *PTEN*, or *Foxo^+^* alone were expressed in motor neurons (Figure 3). Thus, eliminating *Foxo* reverses the hyperexcitability conferred by blocking PI3K pathway in motor neurons, whereas overexpressing *Foxo^+^* reverses the hypoexcitability confered by activating PI3K in motor neurons. These epistasis tests support the notion that PI3K activity decreases motor neuron excitability by inhibiting Foxo.

**Figure 4:**
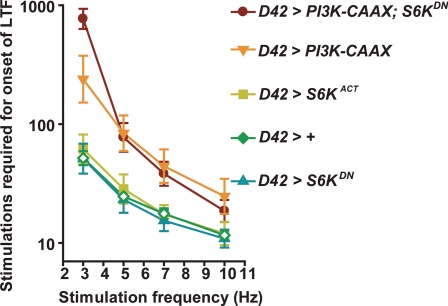
*S6K* does not mediate the effects of *PI3K* on motor neuron excitability. Number of stimulations required to induce LTF (Y axis) at the indicated stimulus frequencies (X axis). The bath solution contained 0.15 mM [Ca^2+^] and 0.1 mM quinidine. Geometric means+/−SEMs are shown. From *top* to *bottom*, *n* = 12, 7, 9, 14, and 18 respectively, for each genotype.

In contrast, we found that altering the Tor/S6K pathway had little effect on motor neuron excitability. In particular, motor neuron expression of neither the dominant-negative *S6K^DN^* nor the constitutively active *S6K^Act^* transgene [39] had any effect on the rate of onset of LTF (Figure 4). In addition, except for the appearance of some enhancement at the lowest stimulus frequency applied, expression of *S6K^DN^* had no effect on the ability of *PI3K-CAAX* to decrease the rate of onset of LTF (Figure 4). Furthermore, expression of *S6K^DN^* had no effect on basal transmitter release, and did not affect the ability of *PI3K-CAAX* to depress basal transmitter release (data not shown). Therefore we conclude that the Tor/S6K pathway does not mediate the effects of PI3K on neuronal excitability.

### The Effects of PI3K on Synapse Number Are Mediated by Tor/S6 Kinase, Not Foxo

**Figure 5:**
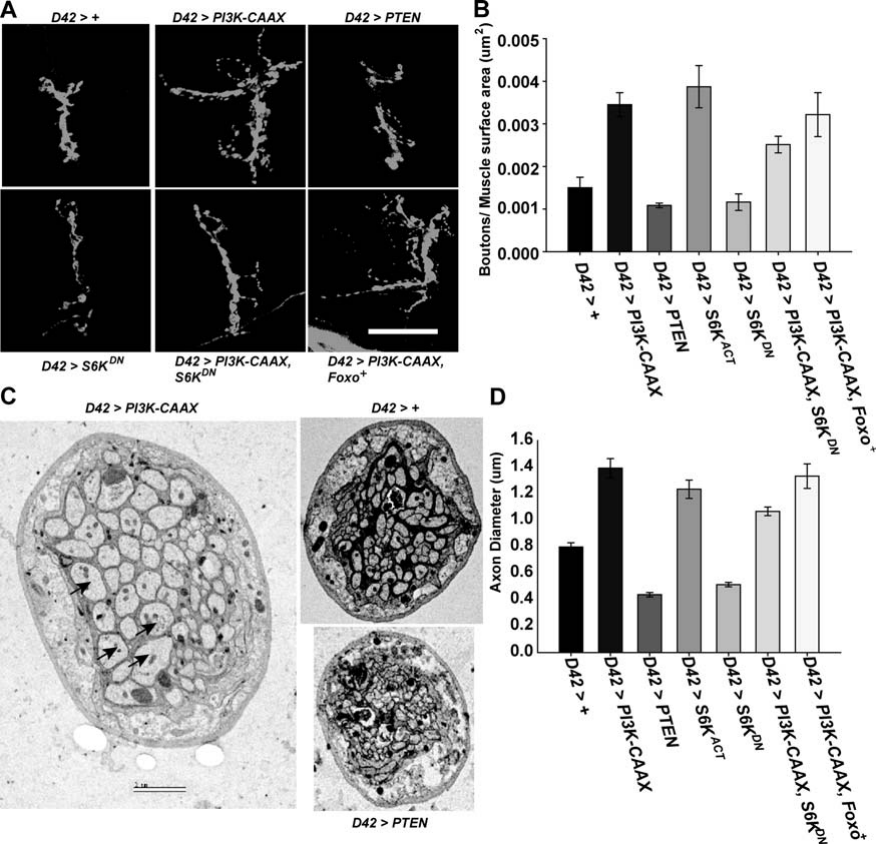
PI3K regulates synapse formation and axon growth via S6K, not Foxo. A) Representative images of muscles 7 and 6 in the indicated genotypes. Larva were stained with anti-HRP (green). Scale bar = 50 µm. B) Mean number (+/−S.E.M.s) of synaptic boutons normalized to the surface area of muscle 6 at abdominal segment A3 in the indicated genotypes. From left to right, n = 6, 8, 6, 6, 7, 11, 11, respectively, for each genotype. One-way ANOVA and Fisher's LSD gave the following differences: For *D42>S6K^Act^* vs. *D42>PI3K-CAAX*, p = 0.40; vs. *D42>+*, p = 0.0009. For *D42>PI3K-CAAX* vs. *D42>PI3K-CAAX*, *Foxo*, p = 0.64; vs. *D42>PI3K-CAAX*, *S6K^DN^*, p = 0.05. C) Representative transmission electron micrographs of peripheral nerve cross sections. Axons are marked with arrows. Scale bar = 2 µm. D) Mean axon diameter (+/−S.E.M.s) of the five largest axons from five different nerves (25 measurements total) for the indicated genotypes. One-way ANOVA and Fisher's LSD gave the following significant differences: for *D42>+*: vs. *D42>PI3K-CAAX*, p<0.0001; vs, *D42>PTEN*, p<0.0001; vs. *D42>S6K^DN^*, p = 0.0009; vs. *D42>S6K^act^*, p<0.0001; vs. *D42>PI3K-CAAX*, *Foxo*, p<0.0001; vs *D42>PI3K-CAAX*, *S6K^DN^*, p = 0.0011. For *D42>PI3K-CAAX* vs. *D42>PI3K-CAAX*, *S6K^DN^*, p = 0.0002. Means from *D42>PI3K-CAAX* and *D42>PI3K-CAAX*, *Foxo* were judged to be not significantly different (p = 0.43).

Because altered PI3K pathway activity alters motor neuron arborization and synapse number [26], it seemed possible that a causal relationship existed between the PI3K-mediated excitability and neuroanatomy defects. To test this possibility, we evaluated the roles of the Tor/S6K and Foxo pathways in mediating the effects of altered PI3K activity on synapse number. We found that motor neuron-specific expression of *S6K^Act^* increased synapse number to an extent similar to *PI3K-CAAX*, and motor neuron expression of *S6K^DN^* partially suppressed the increase in synapse number conferred by *PI3K-CAAX* (Figure 5A and 5B). These observations suggest that S6K mediates in part the effects of PI3K on arborization and synapse number. However, the ability of S6K^DN^ to suppress only partially the effects of PI3K-CAAX overgrowth suggests that both Tor/S6K and a second, PI3K-mediated, pathway (presumably involving GSK3) regulate synapse formation. A role for the Tor/S6K in the control of synapse number was previously reported by Knox et al. (2007). In this report, null mutations in *S6K* decreased synapse number as well as muscle size at the larval nmj. However, it was further reported that activation of the PI3K effector Rheb, which activates Tor/S6K, increased synapse number at the larval nmj even when Tor activity was inhibited by rapamycin [40], raising the possibility that Rheb activates synapse formation via multiple redundant pathways, including Tor/S6K.

In contrast to the effects of altered S6K on synapse formation, we found that *Foxo^+^* overexpression had no effect on synapse number (data not shown) and failed to suppress the growth-promoting effects of PI3K-CAAX (Figure 5A and 5B).

We found that the PI3K pathway also affects axon diameter. In Drosophila peripheral nerves, about 80 axons, including about 35 motor axons, are wrapped by about three layers of glia, as shown in the transmission electron micrograph from cross sections of peripheral nerves in Figure 5C. We found that motor neuron specific expression of *PTEN* decreased motor axon diameter, whereas motor-neuron specific expression of *PI3K-CAAX* increased motor axon diameter. Tor/S6K, but not Foxo, mediates this growth effect. In particular, motor neuron-specific expression of *S6K^Act^* increased axon diameter to an extent similar to *PI3K-CAAX*, and motor-neuron-specific expression of *S6K^DN^* decreased motor axon diameter to an extent similar to *PTEN* and also partially suppressed the growth-promoting effects conferred by *PI3K-CAAX*. In contrast, *Foxo^+^* overexpression did not have a significant effect on the ability of PI3K-CAAX to increase axon diameter (Figure 5D). Therefore, Foxo mediates the excitability effects, but not the growth-promoting effects, of altered PI3K pathway activity, whereas the Tor/S6K pathway mediates in part the growth promoting effects but not the excitability effects of altered PI3K pathway. We conclude that the excitability and growth effects are completely separable genetically and thus have no causal relationship.

### Activity-Dependent Increase in Synapse Number Requires PI3K Activity

**Figure 6:**
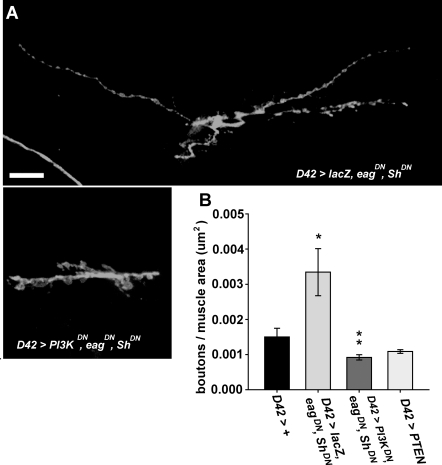
*PI3K^DN^* expression suppresses the synaptic overgrowth conferred by motor neuron expression of *eag^DN^* and *Sh^DN^*. The *D42 Gal4* driver was used to induce motor neuron transgene expression. A) Representative confocal images of muscles 7 and 6 in the indicated genotypes. Larvae were stained with anti-HRP (green). Scale bar = 20 µm. B) Mean number (+/−S.E.M.s) of synaptic boutons normalized to the surface area (Y axis) of muscle 6 at abdominal segment A3 in the indicated genotypes (X axis). From *top* to *bottom*, *n* = 12, 6, 6 and 6 respectively, for each genotype. One-way ANOVA and Fisher's LSD gave the following differences: For *D42>lacZ*, *eag^DN^*, *Sh^DN^* vs. *D42>+*, p = 0.027; vs. *D42>PI3K^DN^*, *eag^DN^*, *Sh^DN^*, p = 0.0075. For *D42>PI3K^DN^*, *eag^DN^*, *Sh^DN^* vs. *D42>PTEN*, p = 0.86.

Depending on the system, neuronal activity can either restrict or promote synapse formation [41]. The Drosophila *eag Sh* double mutant, in which two distinct potassium channel subunits are simultaneously disrupted, displays extreme neuronal hyperexcitability [25], and a consequent increase in synapse number [42],[43]. This activity-dependent increase in synapse number does not require DmGluRA activity [16], suggesting that excessive glutamate release is not necessary for this excessive growth to occur. To determine if PI3K activity is required for this overgrowth, we compared synapse number in wildtype larvae, in larvae expressing dominant-negative transgenes for both *eag* (*eag^DN^*) and *Sh* (*Sh^DN^*) [44],[45] in motor neurons, and in larvae co-expressing *eag^DN^*, *Sh^DN^* and *PI3K^DN^*. We found that co-expression of *eag^DN^* and *Sh^DN^* in motor neurons increased synapse number similarly to what was observed previously [42], and that this increase was completely blocked by simultaneous expression of *PI3K^DN^* but not by *lacZ* (Figure 6). Thus, the activity-dependent increase in synapse formation requires PI3K activity. The observation that glutamate activation of DmGluRA is not necessary for this increase raises the possibility that another PI3K activator contributes to synapse formation at the larval nmj. Insulin is a plausible candidate for such an activator because both insulin and insulin receptor immunoreactivity are present at the nmj [46].

## Discussion

### A Mechanism for the Glutamate-Induced Negative Feedback of Motor Neuron Excitability

**Figure 7:**
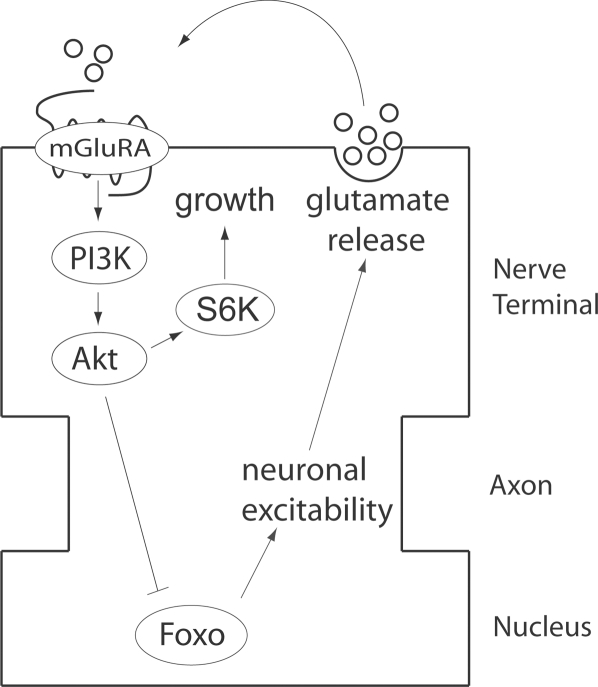
A model for the negative feedback loop regulating motor neuron excitability. The transcription factor Foxo increases neuronal excitability through a mechanism possibly involving transcription of ion channel subunits or regulators. This increased excitability promotes glutamate release from motor nerve terminals, which then activates presynaptic DmGluRA in an autocrine manner. This activation, in turn, activates PI3K and the subsequent inactivation of Foxo by Akt-mediated inhibitory phosphorylation. Activated PI3K also promotes axonal growth and synapse formation via the Tor/S6K pathway.

The effects on neuronal excitability of altered DmGluRA, PI3K, and Foxo activities are consistent with a model in which glutamate released from motor nerve terminals as a consequence of motor neuron activity activates motor neuron PI3K via DmGluRA autoreceptors, which then downregulate neuronal excitability via inhibition of Foxo (Figure 7). Foxo, in turn, might regulate excitability via transcription of ion channel subunits or regulators. Although such putative Foxo targets have not been identified, one potential target might be the translational repressor encoded by *pumilio (pum)*: *pum* expression is downregulated by neuronal activity, Pum decreases transcript levels of the sodium channel encoded by *para*, and both *para* overexpression and *pum* mutations increase rate of onset of LTF in a manner similar to that described here [19],[21],[23],[24].

### Other Negative Feedback Systems at the Drosophila nmj

The DmGluRA-dependent negative feedback reported here co-exists with several other negative feedback systems operating at the Drosophila nmj. In addition to altered excitability, these systems include alterations in the vesicle release properties of the motor nerve terminal and density of the muscle glutamate receptors [1]. Presumably, these diverse feedback systems, acting in parallel, regulate specific aspects of neuronal function. The DmGluRA-dependent feedback system reported here differs in several respects from some of the other feedback systems reported. For example, this DmGluRA-dependent feedback apparently involves transcriptional changes, suggesting that this system operates on a long time scale and thus will be responsive to chronic, rather than acute, changes in neuronal activity. In addition, this system is likely to be motor neuron-cell autonomous, and will not involve participation of additional cells, such as target muscles or adjacent glia. Furthermore, this system is predicted to link mechanistically several PI3K-dependent processes, including activity-dependent downregulation of neuronal excitability and upregulation of neuronal growth. In this regard, the PI3K-dependent inhibition of Foxo might protect neurons from excitotoxic effects of prolonged stimulation; such protection would not be accomplished by the other feedback systems operating.

### Role of Mammalian mGluRs and PI3K in Regulation of Ion Channel Activity

Both mGluRs and PI3K have been previously implicated in regulation of ion channel activity. For example, ligand activation of group I mGluRs trigger Gαq-mediated release of Ca^2+^ from intracellular stores, and consequently activate Ca^2+^-dependent K^+^ channels and nonselective cation channels [47], whereas activation of group II mGluRs inhibit transmitter release via inhibition of P/Q Ca^2+^ channels [48],[49]. PI3K activation can promote ion channel insertion into cell membranes [50]–[52] and can mediate the decrease in excitability conferred by application of leptin, the product of the obese gene, by activating Ca^2+^-dependent K^+^ channels [53]. However, to our knowledge, effects of mGluR or PI3K activation on ion channel transcription in the nervous system have not been reported.

PI3K also regulates ion channel activity in non-excitable cells. PI3K mediates the ability of insulin growth factor to activate the Eag channel, and the ability of serum to activate the intermediate-conductance Ca^2+^-activated K^+^ channel in breast carcinoma and lymphoma cells, respectively [54],[55]. Interestingly, in these non-excitable cells, activation is accomplished by both acute effects on channel activity as well as long term effects as a consequence of increased channel transcription. Therefore, PI3K can regulate channel activity over different time courses, and via distinct mechanisms, presumably via distinct effector pathways.

### Neuronal Excitability in Human Disease

Human orthologues of group II mGluRs and PI3K are implicated in several neurological disorders. For example, group II mGluRs are potential drug targets for schizophrenia, epilepsy, and anxiety disorders [12]–[14], raising the possibility that altered excitability of glutamatergic neurons might play a role in these disorders. In addition, levels of phospho-Foxo, a product of PI3K/Akt activity, are increased following induction of seizures in rats, and in the hippocampi of epileptic patients [56]. This activity-induced increase in phospho-Foxo was interpreted as a mechanism to protect neurons from the excitotoxic effects of excessive glutamate release because Foxo is more likely than phospho-Foxo to promote apoptosis. Our results raise the possibility that this increase in phospho-Foxo levels occurs via glutamate-induced PI3K activation mediated by group II mGluRs, and interpret this increase as a negative feedback on excitability. A role for PI3K activity in inhibiting epileptic seizures is further supported by the recent observation that application of leptin, a known PI3K activator, inhibits seizures in a PI3K-dependent manner [57]. Increased insulin/IGF levels and increased PI3K activity are also implicated in autism spectrum disorders [58],[59]. These increases are generally hypothesized to affect neuronal function by increasing arborization and synapse formation, but our results raise the possibility that altered neuronal excitability might also contribute. Thus, the results reported here might have significance for several human neurological disorders.

### A Novel Signalling Pathway Linking Group II mGluRs and PI3K

The mechanism by which glutamate-activated DmGluRA activates PI3K remains unknown. Although mammalian group I mGluRs activate PI3K via the Homer scaffolding protein and the PI3K enhancer PIKE [60], Drosophila mGluRA, similar to mammalian group II mGluRs, lack Homer binding motifs [61] and thus would not be predicted to activate PI3K by this mechanism. Alternatively, although the inhibition of glutamate-induced p-Akt activation by *PI3K^DN^* expression demonstrates that PI3K activity is required for this activation, it remains possible that glutamate increases p-Akt levels by activating an enzyme in addition to PI3K. For example, Akt is reported to be phosphorylated and activated by Calmodulin-dependent kinase kinase [62]. Additionally, glutamate-activated DmGluRA might activate PI3K in motor nerve terminals indirectly by triggering Ca^2+^ release from stores, leading to release of insulin and hence activation of PI3K by well-established mechanisms. Further experiments will be required to address these issues.

## Materials and Methods

### Drosophila Stocks

Fly stocks were maintained on standard cornmeal/ agar Drosophila media at room temperature. *D42* and *OK6* express *Gal4* in motor neurons and were provided by Tom Schwarz, Boston, Massachusetts, and Hermann Aberle, Tubingen, Germany respectively. The *UAS-PI3K^DN^* (D954A) and *UAS-PI3K-CAAX* transgenes were provided by Sally Leevers, London, UK, the *UAS-Foxo^+^* transgene was provided by Marc Tatar, Providence, RI, the *Foxo^21^* and *Foxo^25^* lines were provided by Heinrich Jasper, Rochester, NY, the *UAS-S6K^DN^* and *UAS-S6K^act^* transgenes were provided by Ping Shen, Athens, GA, and the *UAS-DmGluRA-RNAi* and the *DmGluRA^112b^* lines were provided by Marie-Laure Parmentier, Montpelier, France. All other fly stocks were provided by the Drosophila stock center, Bloomington, IN.

### Immunocytochemistry

FITC conjugated antibodies against horseradish peroxidase (HRP) were raised in goat (Jackson ImmunoResearch) and were used at 1∶400 dilution. Antibodies against Drosophila p-Akt (Ser505) were raised in rabbit (Cell Signaling Technologies) and were used at 1∶500 dilution. Rhodamine Red conjugated goat anti-rabbit (Jackson ImmunoResearch) was used at a dilution of 1∶1000. For arborization measurements, larvae were dissected in PBS-T and fixed in 4% paraformaldehyde. Images were taken on a Zeiss 410 laser scanning confocal microscope (LSM) with a 20× objective. ImageJ was used to obtain surface area measurements of muscle 6 from abdominal segment A3, and the number of boutons was counted manually. For p-Akt measurements, larvae were dissected in Grace's insect cell culture media (Gibco). When glutamate was applied,100 µM glutamic acid monosodium salt monohydrate (Acros Organics) dissolved in Grace's insect cell culture media was added to the well of the dissection plate. 1 minute after glutamate addition, larvae were rapidly washed in standard saline (0.128 M NaCl, 2.0 mM KCl, 4.0 mM MgCl_2_, 0.34 M sucrose, 5.0 mM HEPES, pH 7.1, and 0.15 mM CaCl_2_), and then immediately fixed in 4% paraformaldehyde. For the 10 min wash, the larvae were washed in Grace's insect cell culture media and placed on shaker for 10 minutes before fixing. Care was taken to treat all samples identically during this procedure. Images were taken on a Zeiss 510 LSM with a 20× objective. Z-stacks were compiled from 2 µm serial sections to a depth adequate to encompass the entire bouton thickness for each sample (from 8–20 µm). Muscles 7 and 6 from either abdominal segments A3 or A4 were used for measurements. ImageJ software was used to analyze p-Akt intensities. In particular, 2D projections were created using the median pixel intensity from each stack at each coordinate point. Neuronal structures, marked by anti-HRP, were traced using the freehand selection tool and the selection was transferred to the anti-p-Akt image where the mean pixel intensity value was measured. Background was obtained with a selection box encompassing the non-neuronal area of muscles 6 and 7 in the particular abdominal segment, the mean pixel intensity was measured and subtracted from the mean p-Akt pixel intensity.

### Electrophysiology

The *D42* motor neuron driver was used to express transgenes for all experiments, except that the *OK6* driver was used for experiments the *Foxo^21^/Foxo^25^* genotype was included. *OK6* is located on a different chromosome from *Foxo*, which simplifies stock construction. Larvae were grown to the wandering third-instar stage in uncrowded bottles at room temperature and dissected as described (17, 18) in standard saline solution (128 mM NaCl, 2.0 mM KCl, 4.0 mM MgCl_2_, 34 mM sucrose, 5.0 mM HEPES, pH 7.1, and CaCl_2_ as specified in the text). Peripheral nerves were cut posterior to the ventral ganglion and were stimulated using a suction electrode. Muscle recordings were taken from muscle 6 in abdominal sections 3–5. Stimulation intensity (5 V for approximately 0.05 msec) was adjusted to 1.5 times threshold, which reproducibly stimulates both axons innervating muscle cell 6. Recording electrodes were pulled using a Flaming/Brown micropipette puller to a tip resistance of 10–40 MΩ and filled with 3M KCl. LTF and ejp amplitude data are reported as geometric, rather than arithmetic means, because the data show a positive skew. For extracellular recordings of neuronal action potentials, a loop of nerve near the nerve terminal was introduced into a suction electrode and nerve activity recorded with a DAM-80 differential amplifier.

### Electron Microscopy

Larvae were grown to the wandering third-instar stage in uncrowded bottles at room temperature. Dissections and preparation for microscopy were performed as previously described [63]. Nerve cross sections close to (within about 10 µm from) the ventral ganglion were obtained and analyzed. Axon diameter measurements were taken from the five largest axons from five different nerves from at least two different larvae.

## Supporting Information

Figure S1 LTF onset is accompanied by supernumerary action potentials in the peripheral nerve. Simultaneous intracellular recordings from muscle (lower traces) and extracellular recordings from the innervating peripheral nerve (upper traces) in the indicated genotypes in response to 10 Hz nerve stimulation. Responses are shown immediately prior to and immediately following LTF onset. Note that LTF onset in each genotype, indicated by arrows, was accompanied by supernumerary, repetitive firing of axons in the innervating nerve (arrowheads). Bath [Ca^2+^] was 0.15 mM, quinidine concentration was 0.1 mM.
(6.7 MB TIF)

Figure S2 PI3K pathway inhibition increases neuronal excitability. Number of stimulations required to induce LTF (Y axis) at the indicated stimulus frequencies (X axis) in the indicated genotypes. Geometric means+/−SEMs are shown. Bath [Ca^2+^] was 0.15 mM. n = 5 for all genotypes. One-way ANOVA and Fisher's LSD gave the following differences, at 10 Hz, 7 Hz, 5 Hz respectively: For *D42>+*: vs. *D42>PI3K^DN^*, p = 0.027, 0.020, 0.0033; vs. *D42*>Foxo, p = 0.0099, 0.018, <0.0001.
(7.0 MB TIF)
