# Supplementary figures and images for: A PI3-Kinase–Mediated Negative Feedback Regulates Neuronal Excitability
Source: PLoS Genet. 2008 Nov 28;4(11):e1000277. doi: 10.1371/journal.pgen.1000277 (PMC2581892; doi:10.1371/journal.pgen.1000277)

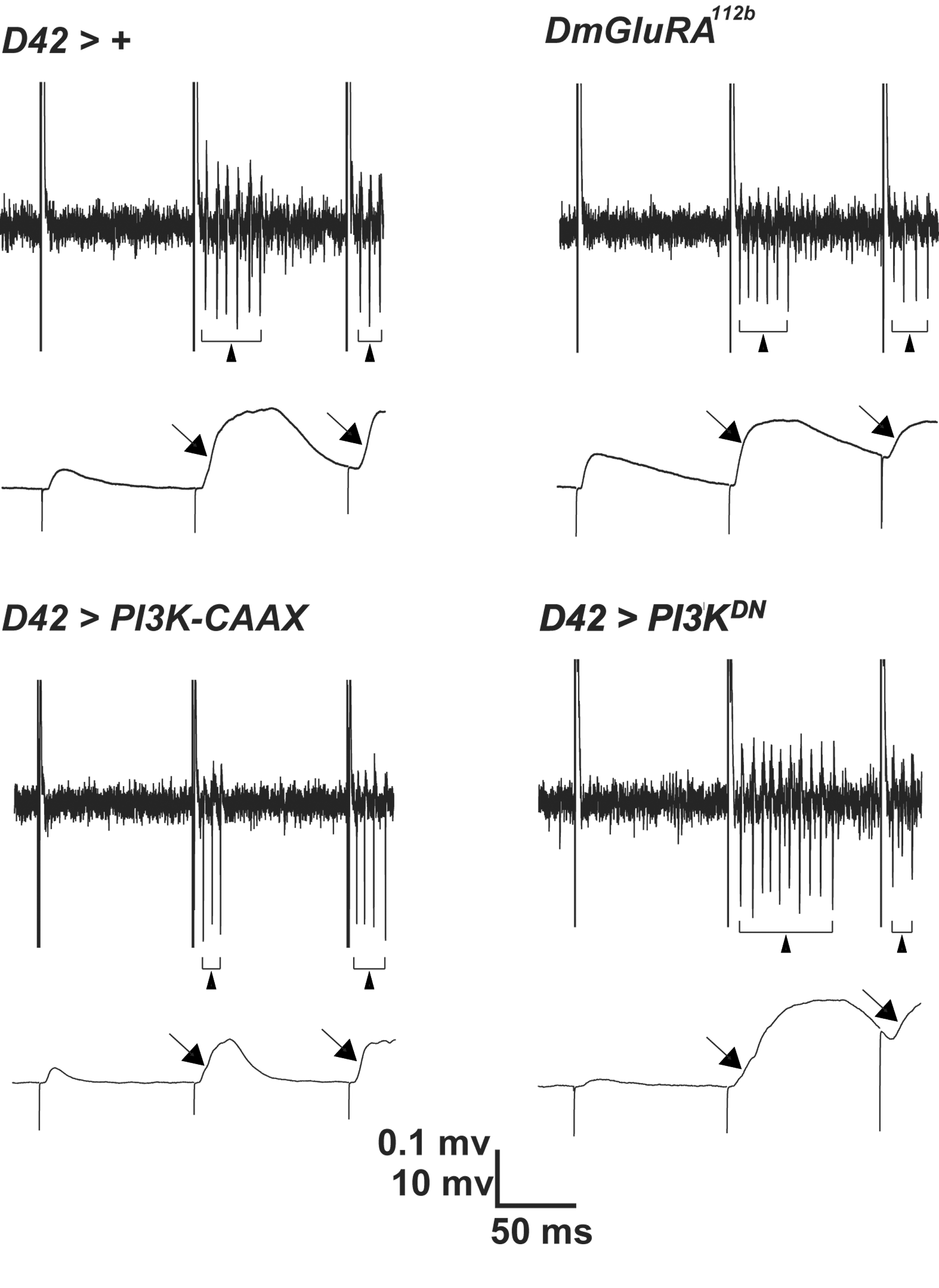

Supplement: Figure S1 — LTF onset is accompanied by supernumerary action potentials in the peripheral nerve. Simultaneous intracellular recordings from muscle (lower traces) and extracellular recordings from the innervating peripheral nerve (upper traces) in the indicated genotypes in response to 10 Hz nerve stimulation. Responses are shown immediately prior to and immediately following LTF onset. Note that LTF onset in each genotype, indicated by arrows, was accompanied by supernumerary, repetitive firing of axons in the innervating nerve (arrowheads). Bath [Ca2+] was 0.15 mM, quinidine concentration was 0.1 mM. (6.7 MB TIF) [file pgen.1000277.s001.tif]

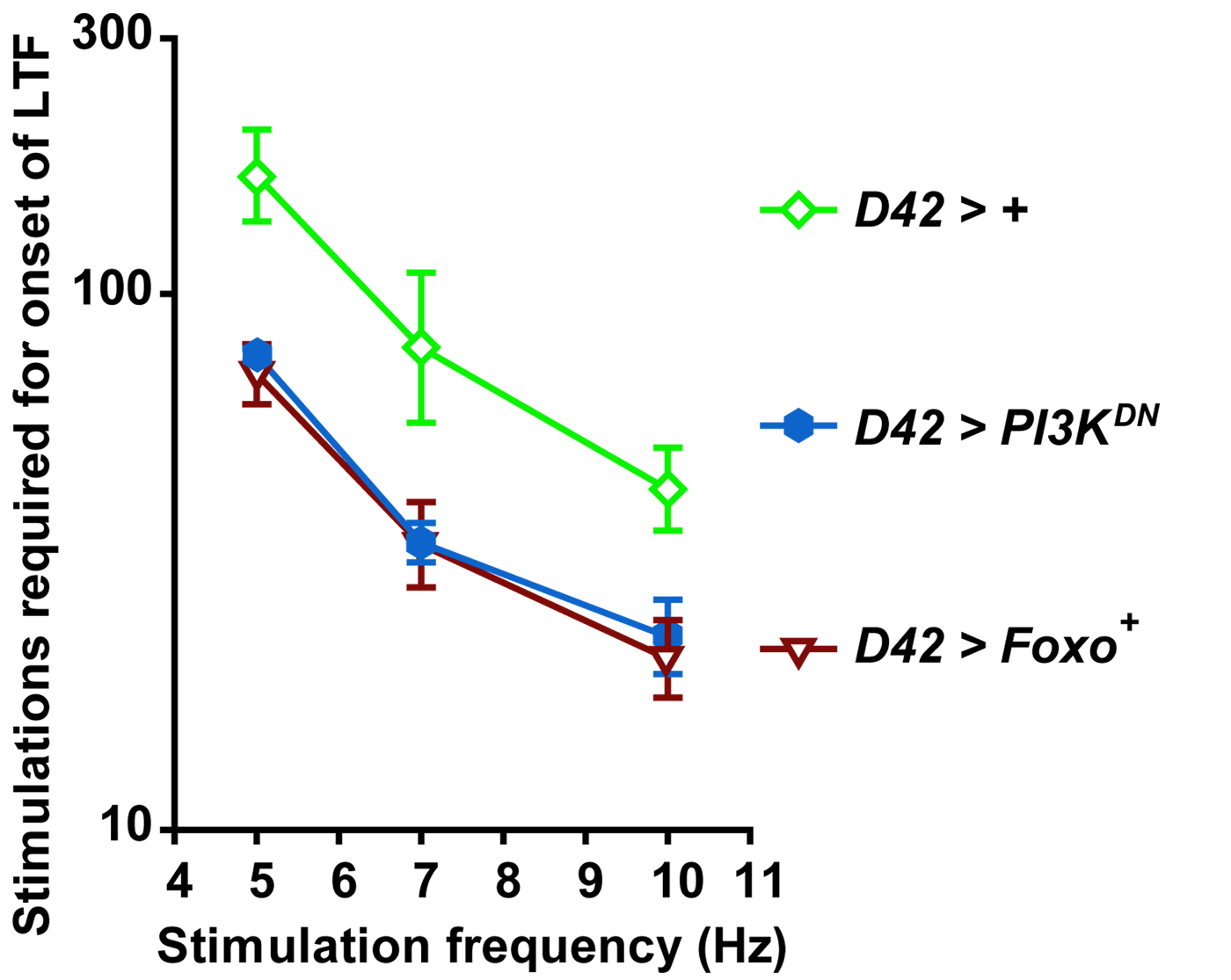

Supplement: Figure S2 — PI3K pathway inhibition increases neuronal excitability. Number of stimulations required to induce LTF (Y axis) at the indicated stimulus frequencies (X axis) in the indicated genotypes. Geometric means+/−SEMs are shown. Bath [Ca2+] was 0.15 mM. n = 5 for all genotypes. One-way ANOVA and Fisher's LSD gave the following differences, at 10 Hz, 7 Hz, 5 Hz respectively: For D42>+: vs. D42>PI3KDN, p = 0.027, 0.020, 0.0033; vs. D42>Foxo, p = 0.0099, 0.018, <0.0001. (7.0 MB TIF) [file pgen.1000277.s002.tif]
